# The Link between Homocysteine and Omega-3 Polyunsaturated Fatty Acid: Critical Appraisal and Future Directions

**DOI:** 10.3390/biom10020219

**Published:** 2020-02-02

**Authors:** Gianluca Rizzo, Antonio Simone Laganà

**Affiliations:** 1 Independent Researcher, Via Venezuela 66, 98121 Messina, Italy; 2 Department of Obstetrics and Gynecology, “Filippo Del Ponte” Hospital, University of Insubria, 21100 Varese, Italy; antoniosimone.lagana@uninsubria.it

**Keywords:** omega-3 polyunsaturated fatty acids, n-3 PUFAs, homocysteine, B vitamins, cardiovascular disease, cognitive decline

## Abstract

Omega-3 polyunsaturated fatty acids and B vitamins are linked to metabolic and degenerative disorders, such as cardiovascular disease and cognitive decline. In the last two decades, the interplay between B vitamins and omega-3 polyunsaturated fatty acids gained increasing attention. Expression control on enzymes involved in the pathway of homocysteine by polyunsaturated fatty acids has been proposed. The methylation process seems crucial for the metabolism of polyunsaturated fatty acids and their distribution within the body. This review summarizes the available data in humans about the link between homocysteine and omega-3 polyunsaturated fatty acids, with a special focus on the meta-analyses of randomized clinical trials. Even if the paucity of available information about the topic does not allow for definitive conclusions, a synergic action between polyunsaturated fatty acids and B vitamins may play a key role in regulating several metabolic pathways. This element could explain a stronger action on homocysteine levels when omega-3 polyunsaturated fatty acids and B vitamins are supplemented simultaneously. To date, a robust rationale of intervention to prevent metabolic diseases is lacking and could be beneficial for individual health and healthcare policy.

## 1. Introduction

Homocysteine (Hcy) is a non-essential and non-coding amino acid containing a thiol group which is synthesized endogenously. It is an intermediate of reaction in the biosynthesis pathway of methionine, an essential amino acid that represents its only dietary precursor [1]. Furthermore, it represents the precursor of cysteine and participates in the recycling pathway of glutathione. Unlike the methionine cycle which is ubiquitous in mammalian cells, the latter metabolic pathway is characteristic of some cytotypes and represents a crucial point of regulation for the physiological role of Hcy [2].

**Figure 1:**
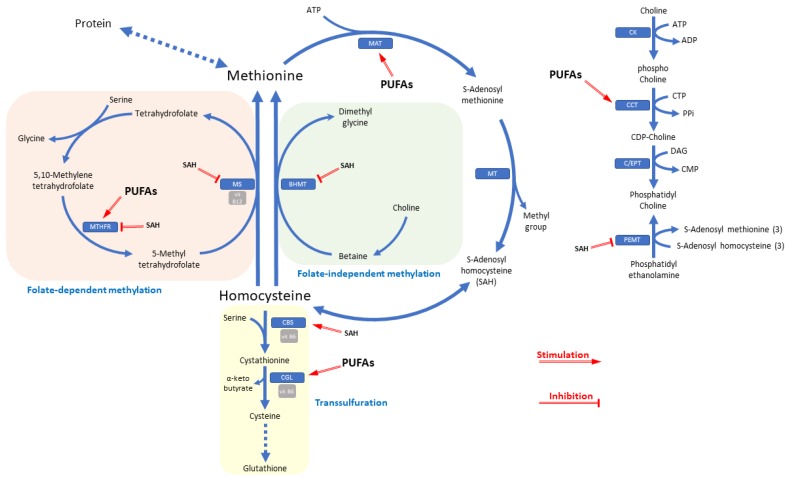
Metabolism of homocysteine and regulation. BHMT: Betaine homocysteine methyltransferase; C/EPT: Choline/ethanolamine phosphotransferase; CBS: Cystathionine-β-synthase; CCT: CTP: choline-phosphatase cytidylyltransferase; CGL: Cystathionine-γ-lyase; CK: Choline Kinase; MAT: Methionine adenosyltransferase; MS: Methionine synthase; MT: Methyltransferase; MTHFR: Methylenetetrahydrofolate reductase; PEMT: phosphatidylethanolamine N-methyltransferase.

When the pool of enzymes responsible for Hcy catabolism is not efficient enough, there is an increase in Hcy molecule concentrations within the cell. Subsequently, the excess of Hcy is eliminated from the cells and its circulating blood levels are increased [3]. This event can occur due to polymorphisms affecting the gene loci of the enzymes of the Hcy pathway (such as methyltetrahydrofolate reductase), medications, and diseases. It can also occur due to insufficiency (even from the diet) of the vitamin cofactors necessary for the catalytic processes, mainly B vitamins (folate, cobalamin, and pyridoxine) [4] whose insufficiency currently represents a social problem worldwide [5]. In particular, folate and vitamin B12 participate in the conversion process of Hcy into methionine, ensuring the recycling of methyl groups, while vitamin B6 participates as a cofactor in two consequent catalytic steps in the transformation of Hcy into cysteine. When these vitamins are not sufficiently available, the pathway is blocked and there is an accumulation of Hcy within the cell (Figure 1) [1].

Folic acid shows the ability to reduce blood Hcy levels [6]. The rise of Hcy concentrations can occur due to dietary insufficiency of folate—which is more common in countries without a mandatory flour fortification program with folic acid—and can also be due to a lack of cobalamin [7,8]. Moreover, cobalamin deficiency is common in infancy, in which the demand for B vitamins is greater due to the biosynthesis processes of *de novo* tissues in senescence [9] and among vegetarians [10]. Numerous pathologies have been related to the rise of Hcy concentrations, such as osteopenia [11] and vascular and neurological diseases [12,13]. Hcy has also gained increasing attention with evidence of its implication in cardiovascular disease (CVD) as an independent risk factor [14,15]. Hyperhomocysteinemia, defined as abnormally high concentrations of total Hcy in blood, could have a causal relationship with these pathologies or represent a consequent metabolic effect with a marker value on the progression of these diseases. Elevated blood concentrations of Hcy have been defined over 15 µmol/L, although a unique consensus for this cut-off is still missing [16]. The link between hyperhomocysteinemia and CVDs has recently been questioned, highlighting that the presence of polymorphisms affecting the enzymes of the Hcy cycle—in particular on the methyltetrahydrofolate reductase gene—may have affected previous results [17,18]. Although uncommon, mutations of genes for cell trafficking proteins of vitamin B12 could also lead to alterations in the metabolism of Hcy [19]. Furthermore, in end-stage renal disease (ESRD) patients are at higher risk of CVDs, which implies 50% mortality, with an incidence from 3 to 45 times higher than in the general population [20,21]. Nevertheless, some recent pieces of evidence suggest that the effect of Hcy concentrations reduction through folic acid intervention does not affect CVD risk reduction [6,22]. 

In addition to the Hcy-lowering treatment with B vitamins, omega-3 polyunsaturated fatty acids (n-3 PUFAs) have also gained attention as a class of nutrients with a possible protective effect on the cardiovascular system [23]. Since the first observation of the protective effect of fatty fish on Eskimo populations, the protective role of n-3 PUFAs has been hypothesized as a preventive/therapeutic strategy for CVDs [24,25]. These are fatty acids containing multiple double bonds between carbon atoms of the molecular scaffold, starting from the third carbon from the terminal methyl group, and which represent a class of essential compounds for humans because of the inability to synthesize them *de novo*. Polyunsaturated fatty acids can be classified in long-chain (lc-PUFAs) or short-chain (sc-PUFAs), depending on the length of the carbon chains and also on the number of unsaturation (double bonds). The most representative compound of n-3 sc-PUFAs is the alpha-linolenic acid (18:3n-3), which can be found in plants that retain the biosynthetic ability. However, mammalians retain the enzymatic pool for the elongation and desaturation reactions to convert n-3 sc-PUFA into n-3 lc-PUFAs such as eicosapentaenoic acid (EPA; 20:5n-3) and docosahexaenoic acid (DHA; 22:6n-3) [26]. For this reason, these latter compounds can only be found in foods derived from animals (and also in some marine algae and seaweeds whose nutritional value is however limited to the production of supplements) [27]. Unfortunately, although the enzymatic pool for the transformation of sc-PUFAs into lc-PUFAs is available in mammals, fish, and birds, the biosynthetic efficiency of this pathway is still debated [28]. Furthermore, since omega 6 is much more represented in nature than omega 3, the pool of elongase and desaturase enzymes is sequestered by the metabolic pathway of arachidonic acid biosynthesis (20:4n-6) starting from linoleic acid (18:2n-6) (n-6 lc-PUFA and sc-PUFA, respectively) [29]. To date, there is no consensus about the adequate bodily levels of EPA and DHA, and there may be different needs at various ages [30]. However, n-3 PUFAs have been found effective for the reduction of cholesterol and triglycerides, which are known to play a key role in CVDs [31,32]. Furthermore, epidemiological data suggest that higher consumption of n-3 PUFAs reduces mortality from CVDs [33].

Recently, some clinical trials have highlighted a possible interaction between Hcy and n-3 PUFAs. Moreover, there is an interest in these two molecules because of their common implications in some pathologies, with relevance to both prevention and treatment [33]. Indeed, cardiovascular diseases, metabolic diseases, and more generally chronic degenerative diseases, represent the main cause of death in the western population [34], but their incidences are also growing among low-income countries and children and adolescents [35]. This represents a critical aspect not only for human health but also for growing health costs [36].

On that basis, the purpose of this review is to highlight the potential relationship between Hcy and n-3 PUFAs through the available data of the literature, focusing on possible mechanisms and relevance in human diseases.

## 2. Methods 

A non-systematic review was done through a search on the PubMed medical search engine (https://www.ncbi.nlm.nih.gov/pubmed/) using the following query:

(homocysteine[Title/Abstract] OR hcy[Title/Abstract] OR hyperhomocysteinemia[Title/Abstract]) AND (“omega-3”[Title/Abstract] OR “n-3”[Title/Abstract] OR “PUFA”[Title/Abstract] OR “n-3pufa”[Title/Abstract] OR “Ω-3”[Title/Abstract] OR “Ω-3pufa”[Title/Abstract] OR “omega-3pufa”[Title/Abstract] OR “epa”[Title/Abstract] OR “dha”[Title/Abstract] OR “fish oil”[Title/Abstract] OR “n-3 fatty acids”[Title/Abstract] OR “ALA”[Title/Abstract] OR “alpha linolenic acid”[Title/Abstract] OR “alpha-linolenic acid”[Title/Abstract] OR “docosahexaenoic”[Title/Abstract] OR “eicosapentaenoic”[Title/Abstract] OR “polyunsaturated”[Title/Abstract]).

Secondary searches were performed using the above-mentioned medical subject headings (MeSH terms) in combination, where available. Other relevant articles were extracted from the reference lists of the articles identified with the primary search engine query.

In the first phase, meta-analyses and systematic reviews were selected. Afterward, more recent references were consulted to complete the available information not covered by papers selected in the first screening phase. The inclusion criteria were as follows: Human studies; observational and interventional studies; the interaction between n-3 PUFAs and Hcy; primary or secondary intervention; dietary or pharmacological doses; only papers written in English. 

## 3. Involvement of Homocysteine in Human Diseases 

Hcy represents an independent predictor of CVDs and all-cause mortality for its role in endothelial health and thrombogenesis [37,38]. However, its role in vascular pathologies does not seem definitively clarified and there are still conflicting data on the usefulness of lowering Hcy levels [39,40,41]. The presence of polymorphisms affecting the gene locus for methylenetetrahydrofolate reductase (MTHFR) could explain why the reduction of Hcy does not always correlate with the reduction of the risk of cardiovascular mortality [17,42,43]. Indeed, the presence of specific genotypes such as MTHFR 677C→T causes the abnormal rise of Hcy concentrations, in particular, if homozygous, with a different response to B vitamins. This phenomenon acts as a confounder in the evaluation of high concentrations of Hcy and cardiovascular risk in observational and interventional studies. 

It is not clear what the exact mechanisms are underlying the correlation between Hcy and diseases, although the involvement of endothelial functions seems plausible [44]. This molecule could directly damage endothelial cells through a mechanism involving the inhibition of nitrogen monoxide (NO) [45], indirectly through the production of reactive oxygen species (ROS) [46], and/or through the perturbation of glutathione-dependent antioxidant activity [47]. NO is a central molecule for endothelial functions, regulating vascular tone, leukocyte migration, and platelet aggregation [48]. Endothelial cells have the enzyme pool for the ubiquitous conversion of Hcy to methionine but also the enzyme pool for the transsulfuration pathway of Hcy into cystathionine (Figure 1). This suggests that Hcy of extracellular origin can adversely affect vascular function, although enzymatic alterations can also be endogenous (and therefore of intracellular origin) when endothelial cells fail to detoxify Hcy [49,50]. So, three forms of tissue-specific hyperhomocysteinemia could be classified: 1) alteration of cystathionine pathway; 2) impairment of methionine cycle (including enzymatic dysfunction and co-enzyme deficiency); and 3) impaired methylation of Hcy betaine-dependent pathway, which is folate-independent [3]. Only the first and third forms could be tissue-specific [2]. An implication on oxidative stress could be suggested by the regulatory mechanism between the remethylation and transsulfuration pathways. Oxidative stress stimulates the activity of the cystathionine-β-synthase enzyme while simultaneously inhibiting the methionine synthase enzyme (Figure 1) [51]. In this way, the transsulfuration pathway is stimulated, which leads to the glutathione regeneration, a central molecule against the oxidative stress. The rise of Hcy can also represent a marker of vitamin deficiency, taking into account that the B vitamins deficiency, especially folate and cobalamin, limits the Hcy pathway that accumulates with other reaction by-products [10]. In this scenario, plasma B vitamins are considered poor markers of vitamin adequacy.

Different diseases can have a different influence on Hcy levels. For example, kidney and vascular diseases are associated with hyperhomocysteinemia [12,52], while insulin resistance and diabetes can cause a reduction [53]. This means that clinical trials on population samples with various illnesses show different interventional outcomes that are not transferable to other diseases or a healthy population. Not surprisingly, this could represent a source of heterogeneity.

## 4. The Role of Omega-3 Polyunsaturated Fatty Acids in Human Diseases 

PUFAs can be found in the phospholipid bilayer of the extra and intracellular cell membranes. The presence of n-3 sc-PUFAs or EPA and DHA in the red blood cell membranes seems to be an indirect marker of individual dietary habits [54]. The protective effect of PUFAs has been widely investigated in observational and interventional studies [32]. The efficacy of n-3 PUFAs could be due (at least in part) to the systemic action of eicosanoids (namely prostaglandins, prostacyclin, thromboxane, leukotrienes, and lipoxins). Eicosanoids are the end products of the physiological pathway of oxidation of PUFAs with an established key role in the inflammation processes [55]. Generally, the production of eicosanoids originating from n-3 PUFAs seems to show an anti-inflammatory, antiplatelet, and vasodilatory effect on the endothelial smooth muscle. These effects are opposite to the eicosanoids derived from n-6 PUFAs through the same pool of cyclooxygenase enzymes [56]. Furthermore, n-3 and n-6 PUFAs compete for engagement of the same enzyme pool, which converts the short-chain precursors of the two series of PUFAs into the long-chain compounds that are substrates for the cyclooxygenase enzymes [29]. The same mechanism occurs with cyclooxygenase enzymes for the production of eicosanoids. Furthermore, the two classes of PUFAs compete for the incorporation into cell membranes [57]. 

Thrombomodulin, von Willebrand factor, and cellular adhesion molecules (like E-selectins), appear to be reduced in endothelial cells in intervention studies using n-3 PUFAs, indicating a role in platelet aggregation [48]. n-3 PUFAs could favor the elimination of free radicals and lower the inflammation through the reduction of C-reactive protein (CRP) [58]. A role of n-3 PUFAs has also been suggested in reducing the processes of adipogenesis and increasing the oxidation of fats, and this could explain the beneficial effect on triglycerides, LDL, and HDL. This could partially affect CVDs [59]. n-3 PUFAs can regulate the levels of CRP in modulation of the inflammatory response through the action of the peroxisome proliferator agonist receptor (PPARs) and the nuclear factor-kB (NF-kB) [60]. In myocytes, eicosanoids appear to play a role in regulating the transmembrane flows of sodium and calcium, thus displaying an antiarrhythmic effect [61]. The anti-inflammatory protective effect of DHA seems to act also at the neuronal level through the action of lipid mediators such as neuroprotectin D1, which modulates cellular signaling through the Akt factor [62]. Recently, the docosanoids have been found to also have a regulatory function in several physiological and pathophysiological processes [63].

## 5. Evidence about the Interaction between Homocysteine and Omega-3 Polyunsaturated Fatty Acids from Systematic Reviews and Meta-Analyses 

In 2004, de Bree and colleagues published a review to identify intervention trials that used n-3 PUFAs and/or B vitamins, to assess their influence on CVDs [64]. The selection of papers included quality controls concerning the randomization, blindness, and the presence of a control group. Of the n-3 PUFAs intervention studies about the effect on Hcy concentration, eight were selected for a total of 441 individuals. Even if no meta-analysis was carried out, it seems the efficacy of n-3 PUFAs alone on Hcy was predominantly null, except for a non-randomized and blinded trial by Olszewski and colleagues carried out in 1993 on 15 males with hyperlipidemia, with 12 g/day of fish oil for three weeks [65]. In another study involving 10 men and 2 women with either healthy or slightly elevated blood lipid levels, Haglund and coworkers achieved a slight reduction in Hcy levels after using a 30 mL/day mixture of fish and primrose oil [66]. Supplementation with fish oil alone did not show significant changes in Hcy. Furthermore, folic acid and vitamin B6 had been administered in both intervention groups. A study of 14 men and 18 women showed a statistically significant rise of Hcy concentrations in the arm involving 6 g/day of fish oil [67]. All other studies did not reach significance for n-3 PUFAs intervention. These results could depend partly on the high heterogeneity of the population sample, n-3 PUFAs doses, and the variable lack of randomization and control. Even blindness, if present, could be affected by the characteristic of the taste of fish oil.

Widely cited, the work of Huang and colleagues (2011) consists of a meta-analysis of randomized, placebo-controlled trials focusing on the effect of n-3 PUFAs on Hcy levels [68]. The analysis involved 11 studies with a total of 352 treated and 355 control individuals. The population sample involved healthy individuals or patients with hyperlipidemia, diabetes, CVDs, peripheral vascular disease, metabolic syndrome, or end-stage renal disease (ESRD). The daily doses administered varied from 0.2 to 6 g per day with a trial duration from 6 weeks to 12 months. The overall weighted mean difference (MD) showed a significant favorable impact of n-3 PUFAs on the reduction of plasma Hcy (MD: −1.59 µmol L; 95% CI: −2.34, −0.83; *p* = 0.0001). The qualitative evaluation of the studies selected using a score method ranged from 0 to 5 was predominantly good with only one study with score 2, two studies with score 3, one study with score 5, and the remaining studies with score 4. Unfortunately, the heterogeneity was significant (I^2^: 52%; *p* = 0.02), due to different sample sizes, doses, duration of the intervention, Hcy at baseline, and study quality. In one of the trials, an increase in Hcy was found in patients with ESRD compared to the healthy population [69]. Heterogeneity lost significance in sensitivity analysis excluding the work of Piolot et al. which involved healthy individuals with increased Hcy after intervention (I^2^: 32%; *p* = 0.15) [67], and even more in the concomitant exclusion of Pilot’s work and the work of Zeman et al. (2006) involving dyslipidemic diabetic individuals (I^2^: 14%; *p* = 0.31) [70]. Heterogeneity could, therefore, depend on the different population samples. MD in the sensitivity analysis did not lose significance. Of the 11 studies, four did not reach statistical significance and only Piolot’s work showed a significant increase in Hcy after treatment.

More recently, Dawson and colleagues (2016) published a meta-analysis of interventional studies on the combined or independent effect of n-3 PUFAs and B vitamins on Hcy levels [71]. Although modest, a negative MD on Hcy level in the treated group compared to placebo was found (MD: −1.09 µmol/L; 95% CI: −2.04, −0.13; *p* = 0.026). Heterogeneity was moderate but significant when PUFAs supplements were used (Model B, I^2^: 57.08%). The result is consistently mitigated compared to the meta-analysis of Huang et al. (2011) mentioned above. The MD for Hcy concentration obtained from datasets with n-3 PUFAs in conjunction with B vitamins was higher (Model C, MD: −1.37 µmol/L; 95% CI: −2.38, −0.36; *p* = 0.008; I^2^: 43.77). Of the 13 studies included in Dawson et al. (2016)’s meta-analysis for the PUFAs-only dataset (1134 treated, 1121 in the control arm), six overlapped with the meta-analysis of Huang et al (2011). A trial reported a rise in Hcy after PUFAs treatment (also present in the meta-analysis of Huang et al., 2011), seven reported unchanged Hcy levels and the remaining five trials reported a reduction. The doses of treatment with n-3 PUFAs ranged from 0.2 to 6 g/day, with a mean study duration of six months (ranging from 2 weeks to 12 months). In the PUFAs plus B vitamins dataset, eight clinical trials were evaluated of which only one study showed a significant difference in treatment compared to control (the SU.FOL.OM3 study) and whose removal from sensitivity analysis makes the final result not significant [72]. Of the eight clinical trials analyzed in this dataset, four overlap with the Huang et al. (2011)’s meta-analysis. The concomitant use of n-3 PUFAs and B vitamins is a confounding factor for the evaluation because of the implication of B vitamins in the Hcy pathway [1]. This implies that Dawson and colleagues (2016) obtained more useful results in understanding the efficacy of n-3 PUFAs alone, compared to Huang et al. (2011)’s meta-analysis which included in the data analysis some clinical trials that used PUFAs in conjunction with B vitamins.

Finally, Xu and colleagues published a meta-analysis on the effect of n-3 PUFAs intervention on indicators of vascular inflammation and serum lipid levels in patients with ESRD [73]. Hcy levels had been assessed as a secondary outcome through the evaluation of three studies (182 treated, 181 controls), with treatment lasting from two to six months. The standard mean difference (SMD) of Hcy for PUFAs treatment showed a reduction using a pooled analysis with a fixed model, with a high heterogeneity (SMD: −0.46; 95% CI: −0.72, 0.20, *p* = 0.001; I^2^: 98.3%, *p* < 0.001), but this result was not significant using the pooled analysis with a random model (SMD: −1.63; 95% CI: −4.24, 0.97, *p* = 0.219; I^2^: 98.3%, *p* < 0.001). However, it should be noted that these are patients with a pathology closely linked to alteration in Hcy concentration and the effect of the treatments could be heavily altered by this element and may not reflect the effect on patients with other pathologies or in the general population. Of the three studies of the pooled analysis for the effect on Hcy, two were in common with Dawson et al. (2016)’s work (model B), of which one was also in common with Huang et al.’s meta-analysis.

Table 1 shows the characteristics of the three meta-analyses mentioned above. 

## 6. Latest Insight and Implications for Cognitive Performance and Neurobiology 

Although interventions with PUFAs and B vitamins to reduce hyperhomocysteinemia have been frequently carried out for the evaluation of CVD outcomes, growing clues suggest their role in pathologies such as cognitive decline and dementia [33,74,75]. There seems to be a close relationship between cognitive status and Hcy levels [76]. A role for PUFAs in age-related cognitive decline was suggested [77,78] and Hcy could be crucial for neurological and vascular pathologies [79], which would explain the relationship between CVD and risk of degenerative and vascular dementia [80]. DHA represents an important constituent in the grey matter cortex in the brain, accounting for 30–40% of fatty acids [81]. Dementia onset could be prevented by n-3 PUFAs intake [74]. A link between Hcy and PUFA in Alzheimer’s disease has been already suggested [82].

In 2014, an observational case-control study by Baierle and colleagues enrolling 12 elderly patients with cognitive decline according to the mini-mental state examination (MMSE) performance test, showed lower levels of serum DHA and total n-3 PUFAs compared to 33 control patients with normal MMSE scores [83]. Cognitive functions were positively associated with DHA and total n-3 PUFAs levels. Furthermore, the case group showed higher total Hcy levels compared to controls (19.92 ± 2.70 vs. 14.67 ± 1.28 µmol/L; *p* <0.05), which correlated significantly and inversely with DHA (r: −0.424, *p* < 0.01), total n-3 PUFAs (r: −0.477, *p* < 0.01;) and positively with n -6/n-3 ratio (r: 0.522, *p* < 0.001).

Jernerén and colleagues (2015) assessed how the baseline status of n-3 PUFAs influenced treatment with B vitamins to decrease Hcy in 168 elderly people (85 treated vs. 83 placebo), with mild cognitive impairment from the VITACOG study, a placebo-controlled trial of Hcy-lowering intervention with B vitamins [84]. The efficacy on brain atrophy rates (about 70% of reduction) was effective only in the upper tertiles for the status of n-3 PUFAs, EPA and DHA in the plasma at baseline but only if Hcy levels were ≥11.3 µmol/L at baseline (p < 0.05). However, the relevance of this analysis should be carefully interpreted due to the small group sizes (ranging from 4 to 23).

In 266 patients aged ≥70 years (133 control and 133 treated) with mild cognitive impairment (MCI), B vitamins treatment for two years slowed cognitive decline [85]. Stratification for n-3 PUFAs tertiles at baseline showed better performances in the upper tertile. Cognitive performances were similar across the tertiles in the placebo group, while the lower n-3 PUFAs tertile at baseline showed no improvement in cognitive decline. Upper n-3 PUFAs tertile had lower levels of Hcy. The sample derived from the VITACOG randomized trial, as for the work of Jernerén et al. [84]. 

In a cross-sectional study with older adults at risk of dementia, the potential link between plasma Hcy and cortical β-amyloid (Aβ) was explored [86]. The authors investigated the role of n-3 PUFAs in the relationship between Aβ and Hcy. There were 177 individuals aged about 75 years included in the study and, although in the adjusted multiple linear regression model plasma Hcy was not associated with cortical Aβ, in the exploratory analysis this association was significant in subjects with low baseline n-3 PUFA index of the erythrocyte membranes (n = 10; b: 0.041; 95% CI: 0.017, 0.066; *p* = 0.005). There was no correlation between Hcy and Aβ in subjects with a high baseline n-3 PUFAs (based on the discriminating value of 4.72% which represents the lower quartile of the sample). Unfortunately, the exploratory analysis sample was very limited. Taking into account that the same group did not find any association between n-3 PUFAs and cortical Aβ in a previous cross-sectional study on elderly with memory alterations [87], the interaction between n-3 PUFAs and Hcy seems to be crucial for the selection of the sample and for the consequent sub-analysis to identify a potential specific association.

## 7. Possible Mechanisms of Interaction between Homocysteine and Omega-3 Polyunsaturated Fatty Acids 

The increase in Hcy due to folate deficiency has been found to be related to a specific production of Reactive Oxygen Species (ROS) that damage the unsaturated chains of PUFAs in human endothelial cell monocultures [46].

PUFAs are highly prone to oxidation because they are highly unsaturated. The generation of oxidized metabolites has historically been considered an adverse event due to the action of mediators of inflammation and oxidative damage on cellular constituents [88]. However, accumulating evidence suggests that the production of eicosanoids by the cyclooxygenase (Cox), lipoxygenases (LOs), and Cytochromes P450 represents a peroxidation process. Products of reaction are autocrine and paracrine chemical mediators which, in the case of those derived from EPA and DHA, mitigate inflammation and reduce the immune response [89]. Furthermore, both the nonenzymatic and enzymatic formation of electrophilic by-products derived from n-3 PUFAs generate bioactive molecules with modulating capacity on nuclear mediators and cell’s signaling pathways. The electrophilic derivates of n-3 lc-PUFAs affect the nuclear factor erythroid 2-related factor 2 (Nrf2), heat shock proteins (HSPs), nuclear factor-kappa B (NF-*k*B) and peroxisome proliferator-activated receptor gamma (PPARγ) actions at multiple levels [90]. These mechanisms have a net effect of reducing oxidative stress and inflammation.

There is a close correlation between the Hcy and lipids, which is evident in the metabolism of phosphatidylcholine (PC) which is added into the VLDL particles [91]. The choline derived from PC is catabolized into betaine which participates as a co-substrate in the methylation of Hcy. DHA acts by stimulating the synthesis of the enzyme CTP:choline-phosphatase cytidylyltransferase (CCT), thus promoting the use of choline in phosphatidylcholine [92].

From the first hypothesis back in 1963 by Mueller and colleagues on the possible influence of the B vitamins on the metabolic pathway of PUFAs [93], the methylation processes, closely associated with B vitamins, seemed to be crucial for the assembly of n-3 PUFAs in phospholipids [94,95]. The key enzyme for the conversion of lc-PUFAs from sc-PUFAs—Δ6-desaturase—is a pyridoxal 5-phosphate (PLP) dependent enzyme. This has been confirmed in human cell culture studies in which the reduction of vitamin B6 perturbed the desaturation process by altering the proportion between n-3 and n-6 PUFA metabolism [96].

PC transports PUFAs and participates in their tissue distribution. When Hcy rises, its precursor S-Adenosylhomocysteine (SAH) accumulates in the cytoplasm. SAH, in turn, inhibits phosphatidylethanolamine N-methyltransferase (PEMT), a key enzyme in PC biosynthesis. There is an inverse correlation between SAH and DHA found in phosphatidylcholine molecules of the erythrocyte from Alzheimer’s disease patients [82]. As a consequence, DHA in plasma PC has been proposed as a potential marker for PEMT activity [97]. In a study of 200 obese children, the composition of PUFAs in the PC of membrane phospholipids of red blood cells showed a lower abundance of EPA and DHA in the presence of single nucleotide polymorphisms borne by the PEMT gene [98]. This phenomenon suggests that the activity of PEMT is intimately linked to the incorporation of specific PUFAs into the PC. Differently, the alternative route of PC synthesis via Cytidine 5-diphosphocholine leads to the prevalent incorporation of saturated fatty acids and medium-chain fatty acids [99].

Hcy can follow the ubiquitous pathway of remethylation into methionine thanks to the enzyme methionine synthase or through the enzyme betaine-homocysteine methyltransferase (BHMT), using methyl group released by betaine derived from the choline catabolism. Betaine acts as a donor of methyl groups and is converted to dimethylglycine (DMG) (Figure 1). Increased DMG concentration has been linked to increased CVD risk [100,101]. It has been hypothesized that the n-3 PUFAs act by modulating the expression of the enzymes involved in the Hcy cycle [102]. DHA appears to regulate the gene expression of cystathionine-γ-lyase, an enzyme that carries out transsulfuration of Hcy in a vitamin B6-dependent catalysis. Furthermore, DHA could up-regulate the synthesis of mRNA of 5-methyltetrahydrofolate reductase enzyme involved in the remethylation pathway to methionine [103]. The rise in the activity of methionine adenosyltransferase (MAT) through the action of PUFAs on the expression of the enzymes’ mRNA, facilitates the metabolization of Hcy by removing it through the action of cystathionine-β-synthase (CBS) [2,3]. By increasing MAT activity, the S-adenosylmethionine (SAM) product of reaction raises and subsequently also SAH which in turn represents an allosteric modulator of the CBS enzyme, stimulating its activity with concomitant inhibition of methylenetetrahydrofolate reductase, betaine-homocysteine methyltransferase and methionine synthase [2,104]. To complete the regulation scheme, n-3 PUFAs seem to modulate mRNA of the cystathionine-γ-lyase (CGL) enzyme, favoring the removal of Hcy and leading to the formation of cysteine [105]. This aspect is particularly relevant taking into account that the transsulfuration of Hcy occurs only in specific tissues such as the kidney, pancreas, liver, lymphocytes, endothelial cells, and small intestine. On the one hand, this represents a key point of regulation between remethylation and transsulfuration pathways [2]. On the other hand, the supply of n-3 PUFAs as fish oil can lead to an increase in NO which in turn inhibits methionine synthase, which would explain the effects of rising Hcy in some intervention trials with n-3 PUFAs [67,106]. This phenomenon suggests that the interaction between Hcy and n-3 PUFAs could have a two-phase dose-response.

Attention towards regulatory mechanisms about covalent modifications of histone proteins, DNA, and other molecules such as microRNAs has recently increased. Such modifications involve biotinylation, acetylation, and methylation [107]. In particular, the methylation of histones and DNA depends on the abundance of donors of methyl groups such as choline and homocysteine cycle reaction intermediates, including methionine, cobalamin, and methyl folate. Indeed, methyl-deficient diets reduce the methylation level of histones H3 and H4 in liver tissue [108]. Hcy, as a sensitive biomarker of one-carbon metabolism, can link epigenetic regulation to some pathological conditions, at least in part. Methylation of DNA and histones allows activation and inactivation of chromatin by a conformational change in euchromatin or heterochromatin, respectively. Hyperhomocysteinemia could accumulate SAH resulting in reduced methylation capacity [109]. Many pathologies such as Alzheimer’s disease, stroke, and atherosclerosis seem to be linked to covalent modifications of DNA and histones induced by hyperhomocysteinemia through epigenetic mechanisms [110]. Interestingly, the oxo-derivatives of the n-3 PUFAs also seem to interact with the histone deacetylases, inhibiting their activity [111,112]. In pregnant women, DHA supplementation acts on the DNA of preterm infants, increasing their methylation [113]. During pregnancy, epigenetic regulation plays a decisive role in fetal programming and so the extra choline necessary for these mechanisms is obtained by stimulating the hepatic expression of the PEMT gene by estrogens with a consequent increase in the synthesis of PC [114].

Figure 1 displays the most important regulation elements discussed in this section.

## 8. Conclusions 

The relationship between Hcy and n-3 PUFAs deserves further investigation. The limited data currently available in the literature does not allow for definitive conclusions about the effectiveness of the treatments for the chronic and degenerative diseases described above, which may be due to the heterogeneity of the studies. In this scenario, further well-designed randomized clinical trials are needed to better understand the underlying mechanisms. It is important to evaluate the 2 × 2 factorial design that allows discriminating the inherent phenomena while simultaneously evaluating the appropriate confounding factors involved. Previously, fish oil was the main substance for interventional studies, but today we can take advantage of more eco-friendly algal extracts that are even easier to standardize in concentrations. One of the challenges is to understand whether the health efficacy of polyunsaturated fatty acids can be obtained with dietary or pharmacological doses. In the observational and interventional studies, the use of more effective tools such as the evaluation of polymorphisms affecting the enzymes involved in the Hcy pathway will be useful. It remains to be determined whether the interaction between n-3 PUFAs and Hcy is independent and/or synergistic with the status of the B vitamins. Likely, their combined use seems to be a more effective approach.

## Figures and Tables

**Table 1:** Effect of omega-3 polyunsaturated fatty acids on homocysteine levels in humans, pooled from meta-analyses of intervention trials.

References	Participants	Trials	Patients	Daily Doses	Duration	Results	95% CI	I^2^
Huang et al. 2011	T: 352; C: 355	11	CHD; ESRD; HL; HT; MI; MS; PVD; T2D	0.2–6 g	6 Wk–12 Mo	MD: −1.59 µmol/L; *p* < 0.0001	−2.34, −0,83	52%; *p* = 0.02
Dawson et al. 2016	T: 1134; C: 1121 T: 787; C: 786	13 8	CVD; ESRD; HL; HT; MI; T2D CHD; CVD; HT; MS; PVD	0.5–6 g 0.2–2 g	2 Wk–12 Mo 3–12 Mo	MD: −1.09 µmol/L; *p* = 0.026 (B) MD: −1.37 µmol/L; *p* = 0.008 (C)	−2.04, −0.13 −2.38, −0.36	57.08%; *p* = 0.006 43.77%; *p* = 0,087
Xu et al. 2016	T: 182; C: 181	3	ESRD	1.7–6 g	2–6 Mo	SMD: −0.46 µmol/L; *p* = 0.001 (Fixed) SMD: −1.63 µmol/L; *p* = 0.219 (Random)	−0.72, −0.20 −4.24, 0.97	98.3%; *p* < 0.001

C: Control; CHD: Coronary heart disease; CI: Confidence interval; CVD: Cardiovascular disease; HL: Hyperlipidaemia; HT: Healthy; ESRD: End-stage renal disease; I2: Heterogeneity test; MD: Mean difference; MI: Myocardial infraction; Mo: Months; MS: Metabolic syndrome; PVD: Perihelial vascular disease; SMD: Standard mean difference; T: Treatment group; T2D: Type 2 diabetes; Wk: Weeks.
